# EUS in the diagnosis of pathologically undiagnosed esophageal tuberculosis

**DOI:** 10.1186/s12876-020-01432-7

**Published:** 2020-08-28

**Authors:** Rong Zhu, Yonghua Bai, Yuankun Zhou, Xingguo Fang, Kui Zhao, Biguang Tuo, Huichao Wu

**Affiliations:** 1 Department of Gastroenterology, Affiliated Hospital, Zunyi Medical University, Zunyi, 563003 China; 2 Department of Pathology, Affiliated Hospital, Zunyi Medical University, Zunyi, 563003 China

**Keywords:** Esophageal tuberculosis (ET), Endoscopic ultrasonography (EUS), Retrosternal pain, Dysphagia

## Abstract

**Background:**

Esophageal tuberculosis (ET) is relatively rare, and the diagnosis is challenging. The aim of this study was to evaluate the clinical features of ET and highlight the role of endoscopic ultrasonography (EUS) in the diagnosis of pathologically undiagnosed ET.

**Methods:**

We retrospectively analysed the clinical features, radiological performances, conventional endoscopic appearances, EUS features, treatment and outcomes of pathologically undiagnosed ET between January 2011 and December 2018. All 9 patients failed to be diagnosed by at least two repeated biopsies (such as routine biopsy, multipoint or deep biopsy, and even or EUS-guided fine-needle aspiration (EUS-FNA)).

**Results:**

Nine patients (66.7% female) with a mean age of 45 years (range 29–59) complained of retrosternal pain or discomfort, or (and) dysphagia. Esophagoscopy demonstrated protruding lesions in the mucosa with central ulcers or erosion in five patients, submucosal bulges with smooth surfaces in one patient, submucosal bulges with diverticula in one patient, ulcers with suspicious fistula formation in one patient, and multiple ulcers in one patient. None of the patients received confirmed histopathological or bacteriological diagnoses by repeated biopsies. However, they were first suspected to have ET based on EUS examination. Because EUS found some characteristic ultrasonographic changes, which were very helpful for the diagnosis of ET when combined with clinical manifestations, the patients subsequently received diagnostic antituberculosis therapy. Finally, the patients recovered or improved with follow-up times ranging from 3 to 10 months.

**Conclusions:**

EUS could help in the diagnosis of ET on basis of EUS features like poorly defined esophageal wall structure, enlarged paraesophageal or mediastinal lymph nodes, hypoechoic lesions of esophageal wall that are linked to the enlarged paraesophageal lymph nodes. However all attempts should be made to obtain histological or microbiological diagnosis.

## Background

Esophageal tuberculosis (ET) is relatively rare, accounting for < 0.2% of all tuberculosis (TB) patients [1–3]. The clinical manifestations are often not specific, including mucosal or submucosal eminence lesions, ulcer, fistula [4], and others [5]. A definite diagnosis of ET requires finding acid-fast bacilli or caseous necrotizing granuloma [6], which is challenging in clinical practice. Therefore, patients with ET are often misdiagnosed or receive a delayed diagnosis. Endoscopic ultrasonography (EUS) can show the shape and echogenicity features of the lesion, its relationship with the esophageal wall, the esophageal wall and extramural changes, such as extramural lymph nodes, and EUS may also provide a cytological and pathological diagnosis by endoscopic ultrasonography-guided fine-needle aspiration (EUS-FNA) [7], or exclude lesions such as cancer. Therefore, EUS has unique value in the diagnosis and differential diagnosis of ET, especially for patients who have not been confirmed by repeated biopsies.

Here, we retrospectively analysed the clinical features and endoscopic and EUS features of ET that failed to be pathologically diagnosed by routine biopsy, multipoint or deep biopsy, and even EUS-FNA in our hospital and summarized the EUS features and their value in the diagnosis of ET. We believe that these features will be significant to clinical work.

## Methods

Nine cases of pathologically undiagnosed ET were identified between January 2011 and December 2018 in the Department of Gastroenterology at the Affiliated Hospital of Zunyi Medical University. These patients did not receive confirmed diagnoses by at least two repeated biopsies (such as routine biopsy, multipoint or deep biopsy, and even EUS-FNA). The patients were retrospectively reviewed for their clinical features, radiological performances, conventional endoscopic appearances, EUS features, treatment and outcomes. All 9 patients were first suspected to have ET based on EUS examination, and when combined with the clinical manifestations, the patients all received diagnostic antituberculosis therapy. Ultimately, the diagnosis of ET was confirmed because the patients recovered or improved, with follow-up times ranging from 3 to 10 months. EUS was performed using a miniature ultrasonic probe (UM-2R 12 MHz; Olympus, Tokyo, Japan) with a circular scanning ultrasound endoscope (UE260-AL5, Olympus, Tokyo, Japan) and a longitudinal scanning ultrasound endoscope (UCT240-AL5, Olympus, Tokyo, Japan). We usually used a miniature ultrasonic probe to observe the changes in the esophageal wall and then used a circular or longitudinal scanning ultrasound endoscope to observe the far-field, such as lymph nodes and surrounding tissues and organs outside the esophageal wall.

## Results

### Clinical features of pathologically undiagnosed ET

Nine patients (66.7% female) with a mean age of 45 years (range 29–59) complained of retrosternal pain or discomfort, or (and) dysphagia, with durations ranging from 2 to 9 wk. Four patients had a history of pulmonary tuberculosis (44.4%). Only two patients had an increased erythrocyte sedimentation rate (ESR) (normal reference value of ESR: 0–15 mm/h for males and 0–20 mm/h for females) (22.2%), but the PPD test or T-SPOT. TB were all positive (PPD test was performed in patients before 2014 and T-SPOT. TB was performed after 2015 in our hospital). Chest CT showed secondary pulmonary tuberculosis in two cases (22.2%), pulmonary fibrotic foci in six cases (66.7%) and multiple calcifications in one case. Eight cases (in case No. 8, biopsy was not performed because the submucosal bulge was small and the surface was smooth) were confirmed by repeated biopsies (3 cases with routine biopsy and deep excavation, 4 cases with routine biopsy and multipoint biopsy, and one case with routine biopsy and EUS-FNA), but the pathological findings were only chronic or acute inflammation, and no specific caseous necrotizing granuloma or acid-fast staining positive bacilli was found. (Table 1).

**Table 1:** Clinical features and Histopathologic findings of undiagnosed ET

Case No.	Age/Sex	Presentation (duration)	Medical history	ESR (mm/h)	PPD test or T-SPOT	Chest CT (or Esophagography)	Histopathologic findings
**1**	41/F	Retrosternal discomfort (4 wk)	Normal	15	PPD(+++)	Pulmonary fibrosis in right lower lobe	Chronic inflammation with suspected epithelioid cells (routine + deep biopsy)
**2**	57/M	Dysphagia (4 wk)	Normal	7	T-SPOT (+)	A few fibrotic foci in the right lung	Chronic inflammation (multipoint biopsy)
**3**	50/F	Dysphagia and retrosternal pain (6 wk)	Pulmonary tuberculosis	30	PPD(++++)	Bilateral secondary pulmonary tuberculosis, with increased mediastinal lymph nodes	Chronic inflammation with acute inflammation (multipoint biopsy)
**4**	35/M	Dysphagia and retrosternal pain (9 wk)	Pulmonary tuberculosis	28	PPD(+++)	Secondary pulmonary tuberculosis in the right upper lobe, with enlargement of mediastinal and left hilar lymph nodes	Chronic inflammation (routine biopsy + EUS-FNA)
**5**	47/M	Retrosternal discomfort (4 wk)	Gastric ulcer	10	T-SPOT (+)	A small amount of proliferation and fibrosis foci in the bilateral lungs	Chronic inflammation with acute inflammation (routine +deep biopsy)
**6**	47/F	Retrosternal discomfort (4 wk)	Pulmonary tuberculosis	6	T-SPOT (+)	A few fibrotic foci in the right lung, right pleural thickening	Chronic inflammation (routine + deep biopsy)
**7**	38/F	Retrosternal discomfort (2 wk)	Normal	5	T-SPOT (+)	A few fibrotic foci in the bilateral lungs (Esophagography: esophageal diverticulum)	Not acquired
**8**	59/F	Dysphagia (4 wk)	Pulmonary tuberculosis	15	T-SPOT (+)	Multiple calcifications in the bilateral lungs and mediastinum, suspicious esophago-mediastinal fistula	Chronic inflammation (multipoint biopsy)
**9**	29/F	Dysphagia (2 wk)	Normal	9	PPD(+++)	A few fibrotic foci in bilateral lungs	Chronic inflammation (multipoint biopsy)

### Esophagoscopy findings and EUS features of pathologically undiagnosed ET

**Fig. 1:**
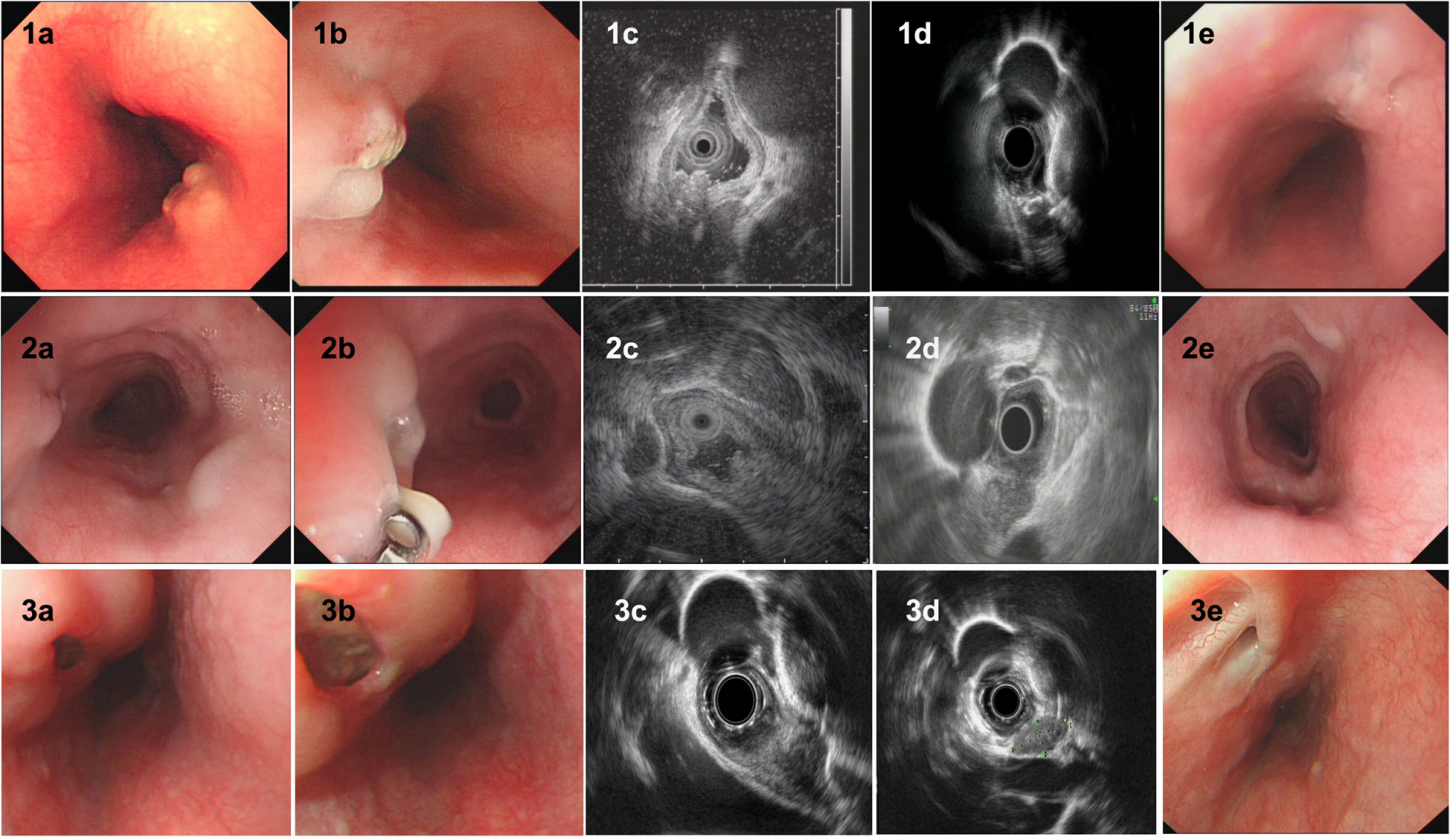
Esophagoscopic findings and EUS features of pathologically undiagnosed ET (cases 1–3). 1–3, case 1–3. **a** (far view) and **b** (near view) Esophagoscopy: a protruding lesion in the mucosa with a central ulcer. **c** and **d**, EUS: thickening, poorly defined esophageal wall structure, with one or more enlarged paraesophageal lymph nodes. **e**, repeat esophagoscopy after treatment: a white mucosal depression with scar formation

**Fig. 2:**
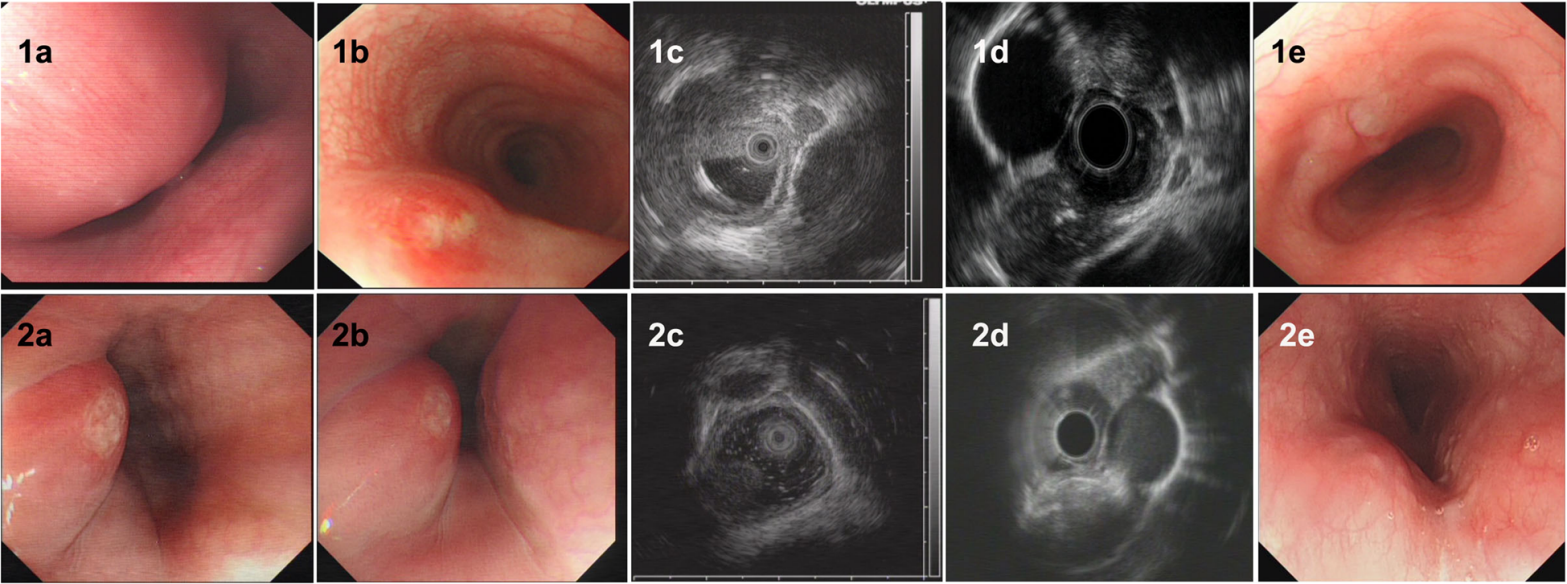
Esophagoscopic findings and EUS features of pathologically undiagnosed ET (cases 4 and 5). 1, case 4; 2, case 5. **a** (far view) and **b** (near view) Esophagoscopy: a protruding lesion in the mucosa with central erosion. **c** and **d**, EUS: thickening, poorly defined esophageal wall structure, with (case 4) or without (case 5) enlarged paraesophageal lymph nodes. **e**, repeat esophagoscopy after treatment: a white mucosal depression with scar formation (case 4) and the protruding lesion in the mucosa became smaller with disappearance of erosion (case 5)

**Fig. 3:**
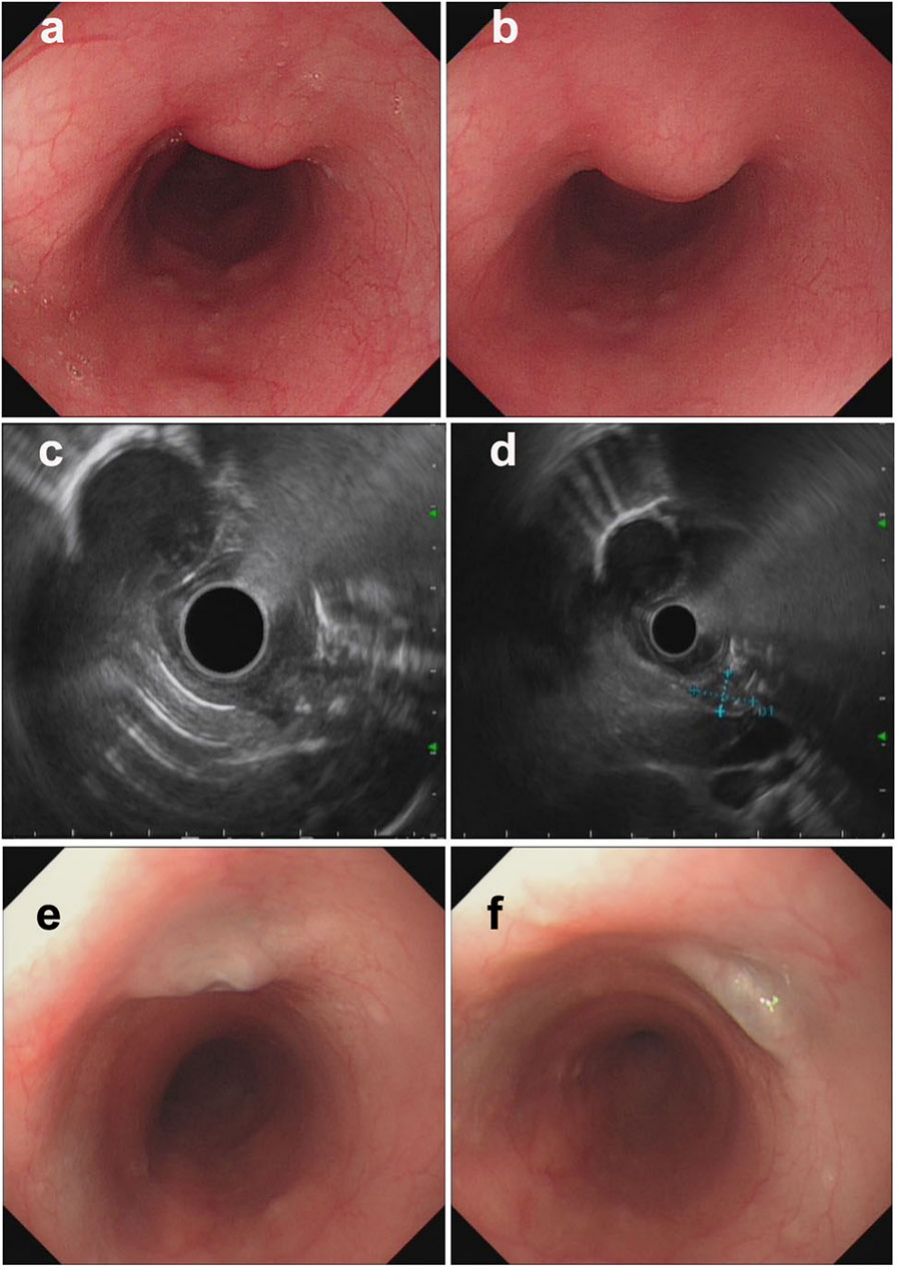
Esophagoscopic findings and EUS features of pathologically undiagnosed ET (case 6). **a** (far view) and **b** (near view) Esophagoscopy: a submucosal bulge with smooth surface. **c** and **d**, EUS: thickening, poorly defined esophageal wall structure, linked to an enlarged paraesophageal lymph node, with scattered calcifications. **e** and **f**, repeat esophagoscopy after treatment: a white mucosal depression with scar formation

**Fig. 4:**
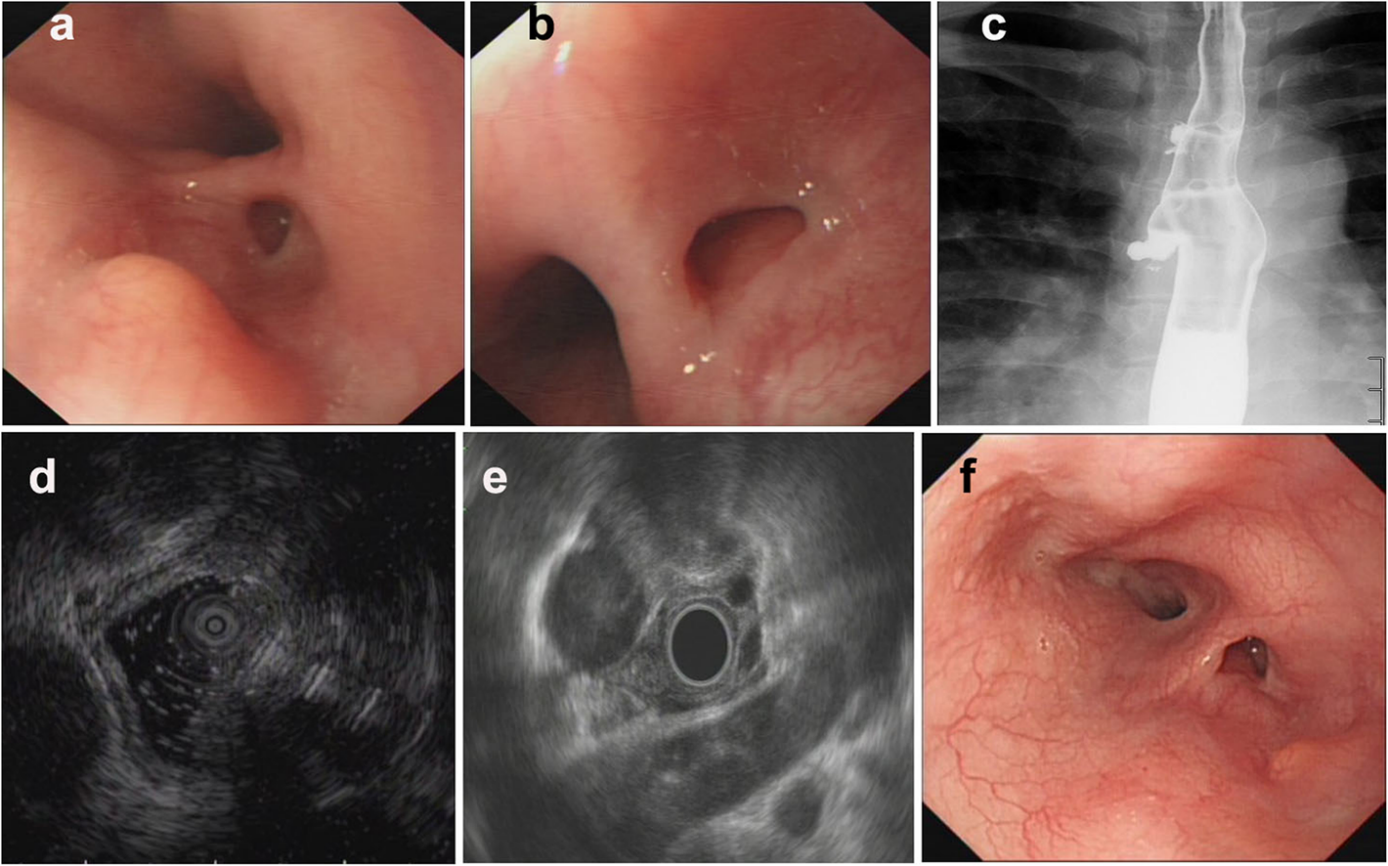
Esophagoscopic findings and EUS features of pathologically undiagnosed ET (case 7). **a** (far view) and **b** (near view) Esophagoscopy: a submucosal bulge and diverticulum. **c**, Esophagography: a diverticulum formation. **d** and **e**, EUS: thickening, poorly defined esophageal wall structure, with enlarged paraesophageal lymph nodes. f, repeat esophagoscopy after treatment: the submucosal bulge became smaller

**Fig. 5:**
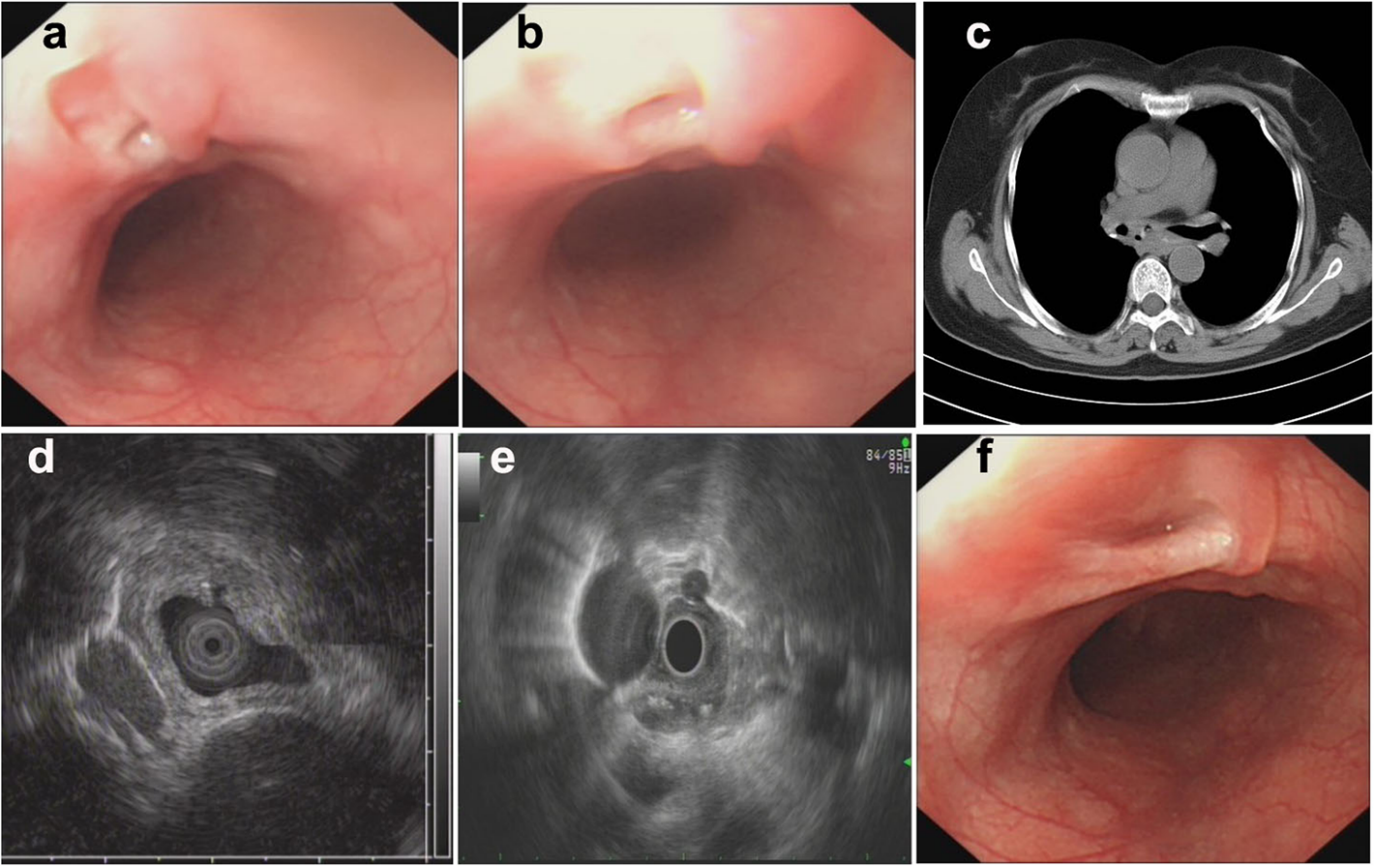
Esophagoscopic findings and EUS features of pathologically undiagnosed ET (case 8). **a** (far view) and **b** (near view) Esophagoscopy: an ulcer with suspicious fistula formation. **c**, chest CT: a small amount of bubbles and fluid in the mediastinum around the esophagus. **d** and **e**, EUS: thickening, poorly defined esophageal wall structure, with one or more enlarged paraesophageal lymph nodes. **f**, repeat esophagoscopy after treatment: a white mucosal depression with scar formation

**Fig. 6:**
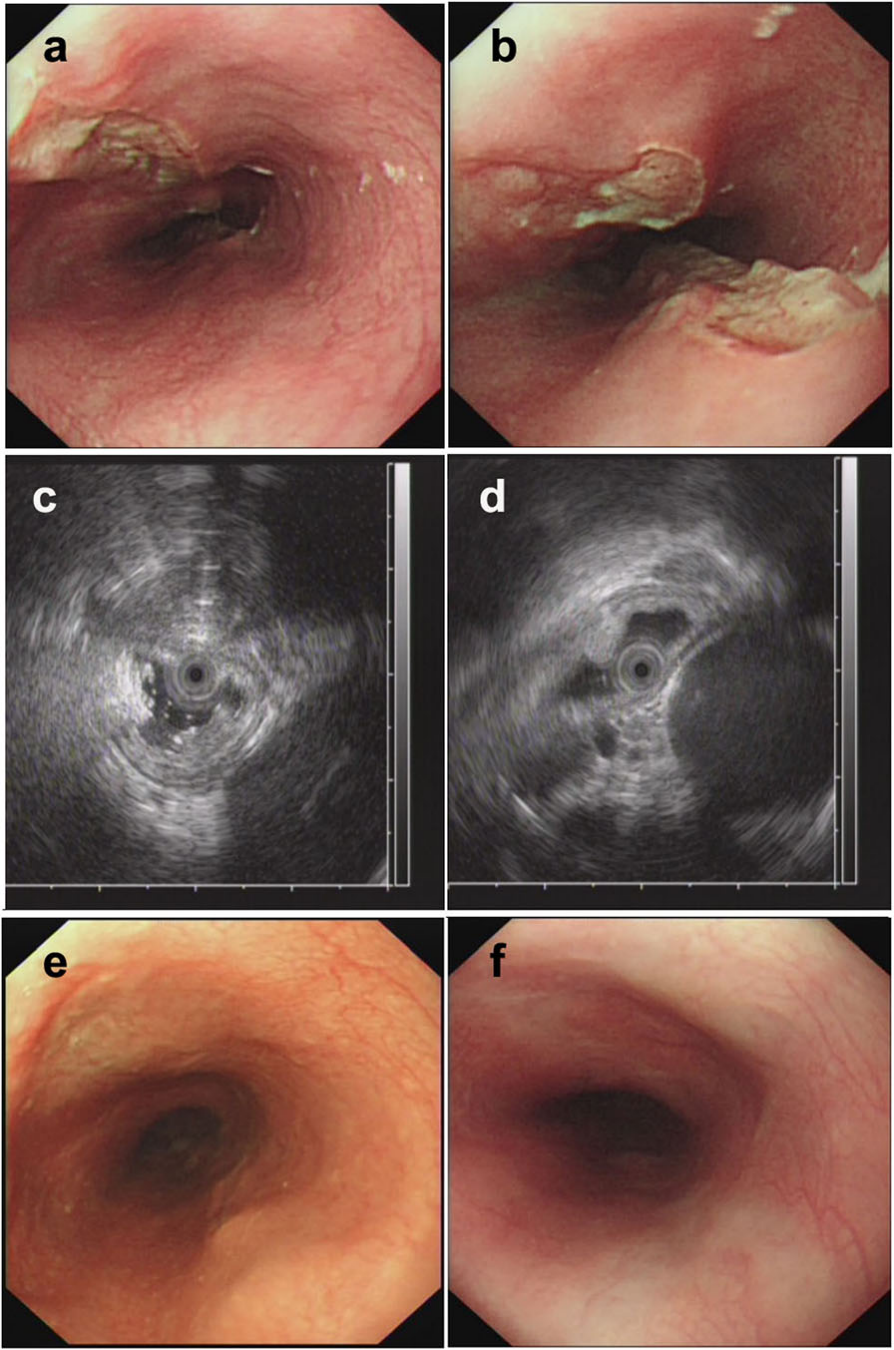
Esophagoscopic findings and EUS features of pathologically undiagnosed ET (case 9). **a** (far view) and **b** (near view) Esophagoscopy: multiple ulcers. **c** and **d**, EUS: Thickening, poorly defined esophageal wall structure, with an enlarged paraesophageal lymph node. **e**, repeat esophagoscopy 2 months after treatment: the ulcers improved. **f**, repeat esophagoscopy 4 months after treatment: white mucosal depressions with scar formation

Of all 9 patients, ET involvement of the esophagus was observed in the middle part in seven cases (77.8%), in the lower part in only one case (11.1%), and in the upper part in only one case (11.1%). The sizes of the main lesions ranged from 0.3 X 0.5 cm to 2.5 X 3.0 cm. Esophagoscopy demonstrated protruding lesions in the mucosa with central ulcers (Fig. 1. 1–3 a and b) or erosion (Fig. 2. 1–2 a and b) in five cases (55.6%), submucosal bulge with smooth surface in one case (11.1%) (Fig. 3. a and b), submucosal bulge and diverticulum (confirmed by esophagography) in one case (11.1%) (Fig. 4. a-c), ulcer with suspicious fistula formation (with a small amount of bubbles and fluid in the mediastinum around the esophagus on chest CT) in one case (11.1%) (Fig. 5. a-c), and multiple ulcers in one case (11.1%) (Fig. 6. a and b). (Tables 1, 2 and Figs.1, 2, 3, 4, 5 and 6).

**Table 2:** Endoscopic findings, EUS features, treatment and prognosis of undiagnosed ET

CaseNo.	Esophagoscopy findings	EUS features	Diagnostic anti- tuberculosis therapy	Prognosis
**1**	Middle part of esophagus, protruding lesion in the mucosa 0.8 X 3.0 cm with a central ulcer 0.3 X 0.5 cm	Thickening, poorly defined esophageal wall structure, enlarged para esophageal lymph nodes with scattered calcifications	Isoniazid + rifampicin + pyrazinamide + ethambutol, 3 months	Recovered according to endoscopy
**2**	Middle part of esophagus, protruding lesion in the mucosa 1.5 X 2.0 cm with a central ulcer 0.2 X 0.3 cm	Thickening, poorly defined esophageal wall structure, linked to an enlarged para esophageal lymph node	Isoniazid + rifampicin + pyrazinamide + ethambutol, 3 months	Recovered according to endoscopy
**3**	Middle part of esophagus, protruding lesion in the mucosa 2.0 X 3.0 cm with a central ulcer 0.5 X 0.8 cm	Thickening, poorly defined esophageal wall structure, with an enlarged para esophageal lymph node	Isoniazid + rifampicin + pyrazinamide + ethambutol, 10 months	Recovered according to endoscopy
**4**	Middle part of esophagus, protruding lesion in the mucosa 2.5 X 3.0 cm with central erosion 0.3 X 0.4 cm	Thickening, poorly defined esophageal wall structure, esophageal heterogeneously hypoechoic lesion with scattered calcifications, and the hypoechoic lesion was linked to an enlarged para-esophageal lymph node	Isoniazid + rifampicin + pyrazinamide + ethambutol, 5 months	Recovered according to endoscopy
**5**	Lower part of esophagus, protruding lesion in the mucosa 1.0 X 1.5 cm with central erosion, 0.2 X 0.2 cm	Thickening, poorly defined esophageal wall structure, with heterogeneously hypoechoic esophageal lesion, and interrupted partial esophageal adventitia	Isoniazid + rifampicin + pyrazinamide + ethambutol, 4 months	Improved according to endoscopy
**6**	Middle part of esophagus, submucosal bulge 0.8 X 1.0 cm with smooth surface	Thickening, poorly defined esophageal wall structure linked to an enlarged para-esophageal lymph node, with scattered calcifications	Isoniazid + rifampicin + pyrazinamide + ethambutol, 5 months	Recovered according to endoscopy
**7**	Upper part of esophagus, submucosal bulge 0.5 X 0.7 cm, and diverticulum	Thickening, poorly defined esophageal wall structure, with enlarged para-esophageal lymph nodes	Isoniazid + rifampicin + pyrazinamide + ethambutol, 9 months	Improved according to endoscopy
**8**	Middle part of esophagus, irregular ulcer 0.3 X 0.5 cm, with fistula	Thickening, poorly defined esophageal wall structure linked to an enlarged para-esophageal lymph node with scattered calcifications	Isoniazid + rifampicin + pyrazinamide + ethambutol, 6 months	Recovered according to endoscopy
**9**	Middle part of esophagus, multiple ulcers 0.3 X 0.5 cm – 0.5 X 0.6 cm	Thickening, poorly defined esophageal wall structure, with an enlarged para-esophageal lymph node	Isoniazid + rifampicin + pyrazinamide + ethambutol, 4 months	Recovered according to endoscopy

Furthermore, the EUS features of pathologically undiagnosed ET were carefully observed and analysed. The results showed that all patients had thickening and a poorly defined esophageal wall structure (100.0%), and the partial esophageal adventitia was interrupted; eight patients had one or more enlarged paraesophageal lymph nodes (88.9%), and four patients had scattered calcifications in the esophageal hypoechoic lesions or paraesophageal lymph nodes (44.4%). Very interestingly, in four patients (cases No. 2, 4, 6 and 8), accounting for 44.4% of the patients, the lesions of the esophageal wall were linked to enlarged paraesophageal lymph nodes, which implies that tuberculosis of the paraesophageal nodes spreads directly to the esophagus wall. (Table 2 and Figs.1, 2, 3, 4, 5 and 6).

### Diagnostic antituberculosis therapy and follow-up of pathologically undiagnosed ET

No patients were confirmed by pathological diagnosis; however, the pathological findings may exclude tumours and provide a reference for subsequent treatment. However, we first considered ET based on EUS examination, and when combined with the clinical manifestations mentioned above, the patients subsequently received diagnostic antituberculosis therapy. After treatment with a quadruple standard dose of antituberculosis drugs (isoniazid + rifampicin + pyrazinamide + ethambutol) (the timing of medical treatment is shown in Table 2), the patients all (100%) recovered (cases 1–4, 6, 8 and 9; Fig. 1. 1-3e, Fig. 2. 1e, Fig. 3 e and f, Fig. 5f and Fig. 6f) or improved (cases 5 and 7, Fig. 2. 2e and Fig. 4f), with follow-up times ranging from 3 to 10 months. Surprisingly, seven patients, accounting for 77.8% of patients, achieved endoscopic recovery within six months, leaving white mucosal depressions with scar formation, which were observed by repeat endoscopy after treatment. (Table 2 and Fig. 1, 2, 3, 4, 5, and 6).

## Discussion

ET is relatively rare. The clinical features of ET are often atypical, so these patients are easily misdiagnosed or often receive delayed diagnoses [8]; additionally, some patients undergo unnecessary esophagectomy, because malignancy cannot be ruled out [9], and some patients are even misdiagnosed with esophageal cancer [10], which causes substantial physical pain and economic losses. In clinical practice, although the diagnostic technology for ET has been improved, some patients with ET still cannot be diagnosed by repeated biopsies. In our hospital from January 2011 to December 2018, there were 23 cases of esophageal tuberculosis confirmed by biopsies (caseous necrotizing granuloma and/or acid-fast bacilli), including 3 cases confirmed by routine biopsy, 6 cases by multipoint biopsy, 12 cases by deep biopsy, and 2 cases by EUS-FNA. However, pathologically undiagnosed ET accounted for 28.1% (9/32) of all cases. The purpose of this study was to summarize the clinical manifestations and EUS features of ET by retrospectively analysing patients with pathologically undiagnosed ET, to improve the understanding of ET and reduce clinical misdiagnosis.

On esophagoscopy, the endoscopic findings of ET are varied, such as mucosal or submucosal eminence lesion, ulcer, diverticulum and fistula formation [11, 12]. In addition, esophagography and chest CT can only provide indirect esophageal images (although chest CT can also visualize changes from pulmonary tuberculosis). Therefore, it is difficult for physicians to judge the disease as ET from the above findings. In addition, none of the patients we discussed received a confirmed histopathological or bacteriological diagnosis by pathology after at least two biopsies (including routine biopsy, multipoint or deep biopsy, and even EUS-FNA). For pathologically undiagnosed ET, EUS has unique advantages because it can show lesions in and outside the esophageal wall, which is especially helpful for differentiation from esophageal cancer. Many patients can finally be diagnosed by EUS-FNA. Esophageal cancer mainly manifest as mucosal lesions, which then invade the submucosa, surrounding lymph nodes and other tissues and organs, while ET is mainly caused by the invasion of tuberculosis into paraesophageal or mediastinal lymph nodes, and some patients have tuberculosis in other parts of the body. Here we summarized the EUS features of ET as follows: (1) thickening, poorly defined esophageal wall structure, and interrupted partial esophageal adventitia; (2) hypoechoic lesion in the esophageal wall, with an irregular boundary and nonhomogeneous inner echogenicity, or with scattered hyperechoic calcifications; (3) enlarged paraesophageal or mediastinal lymph nodes or some with scattered calcifications; and (4) in some patients, lesions of esophageal wall that are linked to the enlarged paraesophageal lymph nodes. The EUS features of ET confirmed by biopsies in our hospital are consistent with those of undiagnosed ET reported in our article. Some of the above EUS features have been reported in the literature (such as heterogeneous or homogeneous hypoechoic masses, interruption of the esophageal adventitia, and enlarged mediastinal lymph nodes [13, 14]). Of the four features, the fourth feature is especially important because it suggests that tuberculosis of the paraesophageal lymph nodes spread directly to the esophagus [15].

In summary, there are three types of mode of involvement of ET according to the method of infection [3, 5]: Type 1, secondary ET, is the most common type and is often secondary to tuberculosis in adjacent organs and tissues, such as paraesophageal or mediastinal lymph node tuberculosis; paraesophageal lesions of the esophagus have often been missed in the pre-EUS era. Puri R et al. [16] showed that ET was commonly secondary to mediastinal lymph nodal involvement in 32 cases of ET, and EUS showed lymph nodes adjacent to esophageal pathology in all cases. In our study, 88.9% (8/9) of patients had one or more enlarged paraesophageal lymph nodes, and 50% (4/8) in the lesions of esophageal wall were linked to enlarged paraesophageal lymph nodes. Therefore, our study focuses on secondary esophageal tuberculosis. Type 2, primary ET, occurs when food or sputum containing *Mycobacterium tuberculosis* is swallowed; *Mycobacterium tuberculosis* adheres to the esophageal mucosa and causes tuberculosis infection, especially when the esophageal mucosa is damaged. Because the esophageal mucosa is composed of squamous epithelial cells covered with saliva and mucus, which have very good protective effects for the esophagus, this type is rare. Type 3, haematogenous disseminated ET, is often seen in patients with severe tuberculosis, and is very rare.

ET may occur in any part of the esophagus, but occurs mainly in the middle part, which is consistent with the literature [17, 18]. The common clinical features are retrosternal pain or discomfort, or (and) dysphagia. Typical tuberculosis poisoning is often absent. Some patients may have a history of tuberculosis in other parts of the body (mostly pulmonary tuberculosis). PPD test or T-SPOT.TB, ESR, chest CT, and esophagography may have certain reference value in the diagnosis of ET [19]. Esophageoscopic findings lack specificity, and pathological biopsies are sometimes disappointing; false-negative results often occur due to the failure to obtain definite caseous necrotizing granuloma and acid-fast bacilli. Therefore, the diagnosis of ET has always been a great challenge in clinical work [20].

For pathologically undiagnosed ET, EUS has the advantage of combining ultrasonography with endoscopy and can show some features of ET. Although biopsies (including EUS-FNA) are sometimes negative, we can first consider the diagnosis of ET based on EUS examination, and exclude tumours though pathology; when EUS features are combined with clinical features, we can consider experimental anti-tuberculosis treatment [19] for ET patients and finally make a definite diagnosis according to treatment effect. In addition, when EUS is used as the initial examination for patients, the characteristics summarized in this paper can be sufficient to help establish our initial diagnosis. However, in clinical work, we should try our best to obtain the gold standard diagnosis (caseous necrotizing granuloma and acid-fast bacilli), and the side effects of anti-tuberculosis drugs should be monitored throughout the course of diagnostic therapy and treatment.

## Conclusions

EUS is an important diagnostic tool for ET, because it can show the lesions in and outside the esophageal wall by endoscopy combined with ultrasonography. In this study, We summarized some significant EUS features of ET, to improve the understanding of ET and reduce clinical misdiagnosis.

## Data Availability

All data analysed during this study are included in this manuscript (Table 1 and Table 2).

## Abbreviations

EUS: Endoscopic ultrasonography
ET: Esophageal tuberculosis
ESR: Erythrocyte sedimentation rate
CT: Computerized tomography
PPD: Purified protein derivative
T-SPOT TB: T cell spot test for tuberculosis infection
EUS-FNA: Endoscopic ultrasonography-guided fine-needle aspiration
